# Proteomic analysis of soybean defense response induced by cotton worm (*prodenia litura*, fabricius) feeding

**DOI:** 10.1186/1477-5956-10-16

**Published:** 2012-03-08

**Authors:** Rui Fan, Hui Wang, Yongli Wang, Deyue Yu

**Affiliations:** 1 National Center for Soybean Improvement, National Key Laboratory of Crop Genetics and Germplasm Enhancement, Nanjing Agricultural University, Nanjing 210095, China; 2 College of Chemical and Material Engineering, Quzhou University, Quzhou 324000, China

**Keywords:** Soybean, Induced resistance, 2-DE, qRT-PCR

## Abstract

**Background:**

Cotton worm is one of the main insects of soybean in southern China. Plants may acquire defense mechanisms that confer protection from predation by herbivores. Induced responses can lead to increased resistance against herbivores in many species. This study focuses on searching changed proteins in soybean defense response induced by cotton worm feeding.

**Results:**

Ten protein spots that are changed in abundance in response to cotton worm feeding were identified by Two-dimensional gel electrophoresis (2-DE). A total of 11 unique proteins from these spots were identified by MALDI-TOF MS. The mRNA and protein relative expression levels of most changed proteins were up-regulated. These proteins were mainly involved in physiological processes, including active oxygen removal, defense signal transduction, and metabolism regulation.

**Conclusion:**

This is the first proteomic analysis of the soybean defense response induced by cotton worm. The differentially expressed proteins could work together to play a major role in the induced defense response. PAL and SAMS were up-regulated at both the protein and mRNA levels. These genes can be strongest candidates for further functional research.

## Background

As an important economic crop, soybean provides significant sources of fatty acids, proteins, vitamins, minerals and other nutrients for humans and animals, and it also has nonfood uses, such as in the production of industrial feedstocks and combustible fuels [1,2]. However, insect pests can adversely affect the yield and quality of soybean. Cotton worm is one of the main insect pests of soybean in southern China [3,4]. Therefore, improvement in resistance to cotton worm is one of the main soybean breeding objectives.

During the process of evolution, plants have acquired defense mechanisms that confer protection from predation by herbivores. Two modes of resistance to herbivores exist: constitutive resistance, which is expressed independent of an attack; and induced resistance, which is activated only after the plant is attacked or otherwise injured [5]. Compared with constitutive resistance, induced resistance may be more durable and compromises plant fitness less by either decreasing further herbivore damage or increasing plant tolerance to herbivory [6]. Induced responses that lead to increased resistance against herbivores have been reported for over 100 species of plants, such as *Arabidopsis *[7-9], tobacco [10], tomato [11,12], rice [13], and soybean [14-16]. The resistance responses induced in plants change with different attackers. For example, the level of resistance induced by soybean looper herbivory with subsequent bean leaf beetle feeding was higher than that induced by bean leaf beetle herbivory with subsequent soybean looper feeding [16]. Resistance induced by the soybean looper, Mexican bean beetle and corn earworm in soybean has been reported in many studies [14-17]. However, induction of resistance in soybean by cotton worm has not been reported.

Many studies have investigated the induced defense response mechanism of plants. The plant hormones salicylic acid (SA), jasmonic acid (JA) and ethylene (ET) are the main players in the regulation of signaling networks involved in induced defense [18-21]. In general, pathogens are more sensitive to SA-dependent responses, whereas herbivorous insects and necrotrophic pathogens are resisted by JA/ET-dependent defenses [22-24]. Ample evidence indicates JA is the main signaling molecule that mediates a plant's defense system against herbivores [19,23]. A classic example is the observation that following attack by *Manduca sexta *larvae, tomato (*Solanum lycopersicum*) leaves accumulate JA, which results in the activation of genes encoding proteinase inhibitor proteins that inhibit digestive Ser proteinases of herbivorous insects and reduce further insect feeding [8,25]. Induced defense response mechanisms also involve the production of defensive compounds, such as proteinase inhibitors that affect insect feeding [25,26], volatile organic compounds that attract parasitoids and predators of the herbivores that feed on the plant [18,27] and extrafloral nectar that arrests carnivorous arthropods on herbivore-infested plants [28].

Proteomics is a powerful tool for the study of protein dynamics, especially in plant stress responses [29,30]. Analysis of protein profiling is important to understand how genes/proteins are regulated. In the present study, we found that soybean resistance to cotton worm increased after induction of resistance by cotton worm feeding. To investigate which proteins are involved in this induced defense response, we used proteomic approaches to provide an overview of the cotton worm-induced defense response in soybean. Ten protein spots showed differential expression patterns in soybean leaves in response to cotton worm feeding, and 11 proteins were identified. Quantitative RT-PCR (qRT-PCR) analysis indicated differentially expressed proteins also showed changes in mRNA expression level.

## Results and discussion

### Induction of soybean defense response by cotton worm feeding

**Figure 1:**
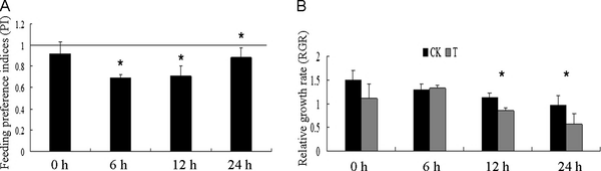
**Identification of induced resistance**. **A**, Feeding preference indices in dual-choice tests.**B**, Relative growth rate of cotton worms fed with control (CK) and treated (T) leaves. Error bars represent the standard deviation. * Significant (*P *< 0.05).

To determine whether cotton worm feeding can induce resistance in soybean leaves, we carried out a dual-choice test and force-feeding experiment (Figure 1). Compared with treated leaves, the cotton worm larvae preferred to eat control leaves, where PI < 1 at the four sampling timepoints (Figure 1A). The relative growth rate of cotton worms feeding on control leaves was higher than those feeding on treated leaves except at 6 h (Figure 1B). In the force-feeding experiment, the relative growth rates of cotton worms feeding on control leaves and those feeding on treated leaves were similar. At this timepoint fifth-instar cotton worms were used, which eat greedily and were not sensitive to the food source [31]. In the dual-choice test and force-feeding experiment, the levels of induced resistance were slightly different at the four sampling timepoints, but these results still indicated that cotton worms can induce resistance in soybean.

Plants do not exhibit induced resistance to insects unless subjected to a certain degree of damage. In pre-experiments, soybean treated with cotton worm feeding shown induced resistance at a damage level of 20-30% and the levels of resistance were not increased significantly when the damage area increased. The capacity for induced resistance showed no difference also at a damage level between 15-45% in mountain birch [32]. However, when leaf damage constitutes 50% of the leaf area, induced resistance was produced in *Quercus garryana *[33]. The timing of induced resistance to herbivory is known to vary between plant species. Usually, in herbaceous plants, the longevity of induced resistance is several days [34-36]. The timing of induced resistance varied with the insect species feeding on soybean. The longevity of resistance induced by *C. trifurcata *was two weeks [16]. The induced response was effective for 3 days after damage against the Mexican bean beetle in soybean. After 15 days, the strength of resistance had declined and by 20 days, all four genotypes exhibited induced susceptibility [37]. In this study, the longevity of induced resistance produced by cotton worm was 5 days.

### Changes of proteins in soybean leaves induced by cotton worm feeding

**Figure 2:**
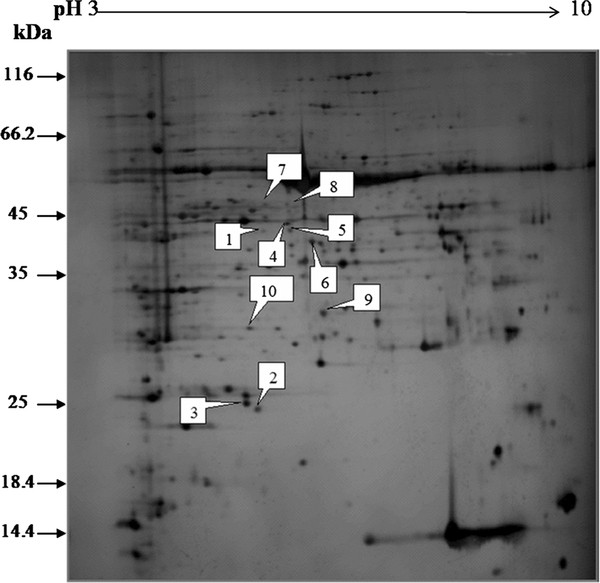
**Representative silver-stained 2D-PAGE gel of separated soybean leaf proteins**. Proteins were separated in the first dimension on a nonlinear IPG strip, pH 3.0-10.0, and in the second dimension on a 12% polyacrylamide SDS-gel. Quantitative image analysis revealed a total of 10 spots that changed in abundance.

**Figure 3:**
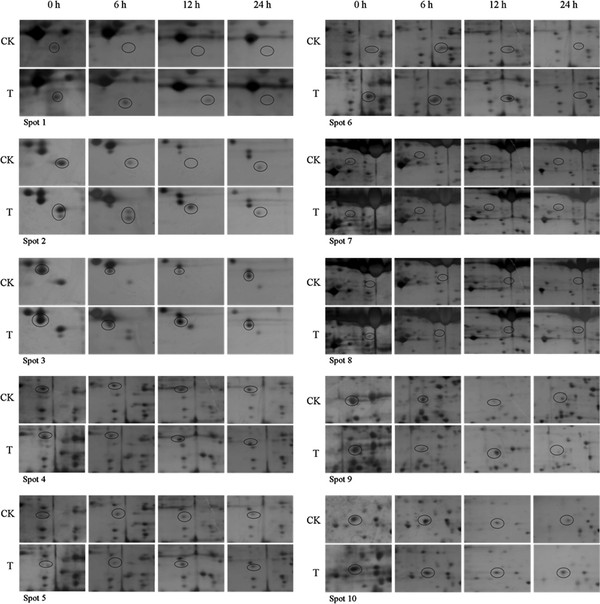
**Enlarged maps of the 10 differentially expressed protein spots**. CK: control; T: soybean leaves treated with cotton worm feeding.

**Figure 4:**
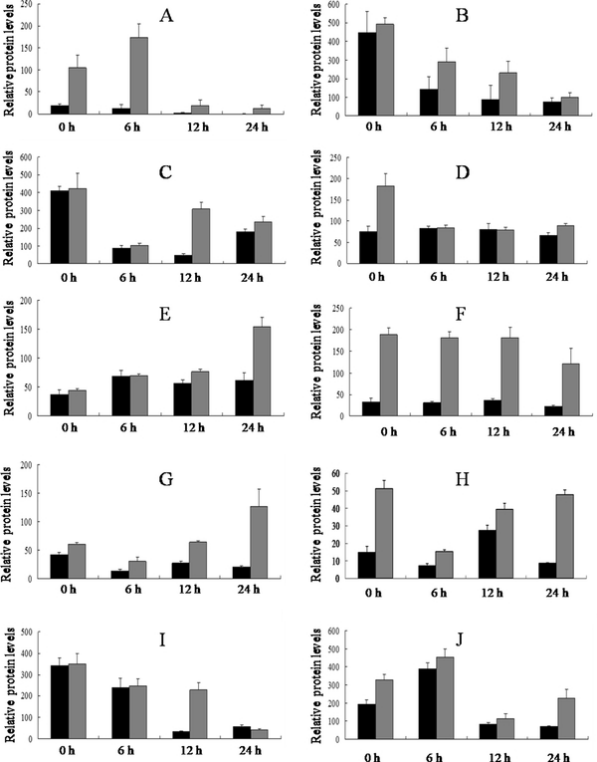
**The volume changes of 10 differentially expressed spots. Control (black) and treated (gray)**. **A - J**represent spot 1 - 10 respectively. Error bars represent the SD. The scales are different for the different proteins.

Soybean leaf proteins extracted 0 h, 6 h, 12 h and 24 h after cotton worm feeding and from the controls were analyzed by 2-DE. Three independent experiments were conducted to ensure that the changes in protein abundance at each timepoint were reproducible and significant. Software quantification showed that, although the protein expression profiles between the treated and control samples were similar, the abundance of some spots changed significantly with cotton worm feeding over time (Figures 2, 3 and 4). Ten spots showed changes in abundance in response to cotton worm feeding, most of which were up-regulated (Figures 2 and 3). The different expression patterns of the spots might imply different roles for these proteins in plant defense responses induced by cotton worm feeding.

### Identification of proteins involved in cotton worm-induced defense response in soybean

All 10 differentially expressed proteins spots were subjected to in-gel digestion and analyzed by MALDI-TOF-MS. The peptide mass fingerprints obtained were used to search the NCBI database using Mascot http://www.matrixscience.com. There were 11 proteins identified from these 10 protein spots (Table 1). Spots 5 and 10 contained two protein species, respectively. Spots 2 and 9 were identified as ribulose-1, 5-bisphosphate carboxylase/oxygenase large subunit. When using 2-DE, co-migrating proteins can present a major problem for quantitative protein expression comparison [38,39]. In this study, real-time RT-PCR was used to help determine which proteins showed altered expression. Occurrence of the same protein in several spots supposedly represents differently modified protein species that might be of biological relevance [38]. Interestingly, the observed Mr/PI of some proteins was different from their theoretical Mr/PI. The reason might result from an unusual posttranslational modification.

**Table 1:** Identification of differentially expressed protein spots by MALDI-TOF MS

Spot no.	Protein name	Mr(KDa)/PI		Sequence coverage (%)	Score	Accession no.
					
		Theoretical	Experimental			
1	serine/threonine kinase-related protein	14.7/9.4	42.0/6.0	46	76	gi|34099884

2/9	ribulose-1,5- bisphosphate	23.3/6.1	24.0/7.7	23	90	gi|157812656
	carboxylase/oxygenase large subunit	51.8/6.2	31.1/6.81	30	172	gi|14599574

3	Vegetative storage protein A	29.2/8.8	24.4/6.1	62	174	gi|134145

4	cytosolic phosphoglycerate kinase	42.3/5.7	43.6/6.4	26	103	gi|9230771

5	ATP synthase beta subunit	50.0/5.7	42.4/6.4	32	81	gi|20269424

5	malate dehydrogenase	36.1/8.2	42.4/6.4	28	68	gi|5929964

6	phenylalanine ammonia-lyase	46.5/6.0	40.3/6.7	56	347	gi|81807

7	S-adenosylmethionine synthetase	42.9/5.4	47.4/6.1	25	88	gi|10443981

8	predicted protein	60.3/5.8	47.0/6.5	24	75	gi|168040478

10	ascorbate peroxidase	27.1/5.7	29.8/5.9	45	110	gi|12229897

10	20S proteasome alpha subunit	27.4/5.8	29.8/5.9	41	104	gi|12229897

**Figure 5:**
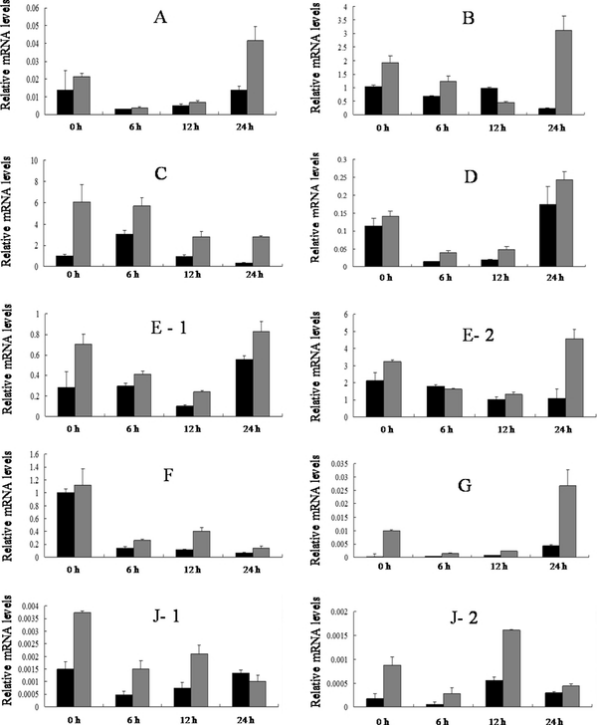
**The expression of 10 differentially expressed spots at the corresponding mRNA level**. **A **represents spot 1.**B **represents spot 2 and 9. **C - G**represent spot 3 - 7 respectively. **J **represents spot 10. **A**, serine/threonine kinase-related protein.**B**, ribulose-1,5- bisphosphate carboxylase/oxygenase large subunit. **C**, Vegetative storage protein **A. D**, cytosolic phosphoglycerate kinase.**E**- 1, ATP synthase beta subunit. **E**- 2, malate dehydrogenase. **F**, phenylalanine ammonia-lyase.**G, S**-adenosylmethionine synthetase.**J**- 1, ascorbate peroxidase. **J**- 2, 20S proteasome alpha subunit. Error bars represent the SD. The scales are different for the different genes.

We verified the cotton worm-induced defense response profile of abundance-changed proteins at mRNA level by real-time qRT-PCR. The mRNA levels of most proteins were up-regulated in response to cotton worm feeding (Figures 4 and 5). ATP synthase beta subunit and malate dehydrogenase were located in the same spot, and the mRNA levels for both proteins were up-regulated, which indicated expression of both protein species in spot 5 was changed in response to cotton worm feeding. Similarly, expression of ascorbate peroxidase and 20S proteasome alpha subunit in spot 10 both changed in response to cotton worm feeding.

Levels of mRNA are not always consistent with the levels of the corresponding proteins [40,41]. Three potential reasons for the lack of a strong correlation between mRNA and protein expression levels have been proposed: (i) translational regulation; Because of many complicated and varied post-transcriptional events, the transcriptome might not always be reflected at proteome levels [42-44]. (ii) differences in protein in vivo half-lives [42,43]; and (iii) significant experimental error, there is a significant amount of error and noise in both protein and mRNA experiments that limit our ability to get a clear picture.

### Differentially expressed proteins

Spot 1 was up-regulated in response to cotton worm feeding, which was identified as serine/threonine kinase (STK)-related protein (Figure 4A and 5A). As a signal protein, STK regulates a variety of cellular processes [45,46]. This study found that STK involved in soybean defense responses induced by cotton worm feeding. Previously, it has been reported that STK was shown to be involved in plant defense responses and induced by low temperature, *Soybean mosaic virus*, and SA [47-49].

Spots 2 and 9 were up-regulated and were identified as ribulose-1,5-bisphosphate carboxylase/oxygenase (RubisCO) large subunit(Figures 4B, I and 5B). RubisCO is the most abundant protein in plants and plays a major role in photosynthesis. RubisCO large subunit is up-regulated under stress, which might be indicative of the protective and more active action of carbon assimilation machinery [50].

Spot 3 was identified as vegetative storage protein α (VSPα). This protein was up-regulated at both protein and mRNA levels (Figures 4C and 5C). The best-characterized VSPs are soybean VSPα and VSPβ VSPs are thought to serve as a transient reserve that sequesters unused amino acids during plant development. Once new seed production begins, stored VSPs presumably make nitrogen and other nutrients immediately available for seed development [51]. This hypothesis, however, was not supported by transgenic soybean in which VSP expression was abolished and seed production was unaffected even under nitrogen-deprived conditions [52]. Thus VSPs may serve other functions beyond source-sink interaction or plant productivity. *Arabidopsis *VSP transcripts are induced by mechanical wounding, JA, insect herbivory, and osmotic and nutritional stresses [9,19,53-55], which is a common response shared by many genes encoding anti-insect proteins. Liu et al. found AtVSP2 is an anti-insect acid phosphatase [56]. Whether soybean VSPα has an anti-insect function needs further study.

Spot 4 showed up-regulation at 0 h and was identified as cytosolic phosphoglycerate kinase (PGK) (Figure 4D). It was up-regulated at all sample timepoints at the mRNA level (Figure 5D). PGK is an enzyme that catalyzes the formation of ATP from ADP and vice versa. In the second step of the second phase in glycolysis, 1,3-bisphosphoglycerate is converted to 3-phosphoglycerate, forming one molecule of ATP. If the reverse occurs, one molecule of ADP is formed. This reaction is essential in most cells for ATP generation in aerobes, for fermentation in anaerobes, and for carbon fixation in plants. Under stress conditions, do plants need to energy has not yet been reported.

Spot 5 was identified as ATP synthase β subunit and malate dehydrogenase (MDH). These proteins were up-regulated at the mRNA level (Figure 5E-1 and 5E-2). ATP synthase is an important enzyme that generates energy for the cell to use through the synthesis of ATP. ATP synthase β was up-regulated in the rice defense response induced by probenazole [39]. MDH is an enzyme in the citric acid cycle that catalyzes the conversion of malate into oxaloacetate (using NAD+) and vice versa (this is a reversible reaction). MDH is not to be confused with malic enzyme, which catalyzes the conversion of malate to pyruvate, producing NADPH. MDH is involved in many physiological activities, such as the organelle shuttle system, plant disease resistance, and metabolism of reactive oxygen species.

Spot 6 was identified as phenylalanine ammonialyase (PAL). This protein was up-regulated at both the protein and mRNA levels (Figures 4F and 5F). It is clear that PAL plays a key role in induced disease resistance based on induction of its expression by pathogens or elicitors, and on studies using transgenic approaches in tobacco [57,58]. PAL is the first enzyme in the phenylpropanoid pathway. In plants, PAL can supply the precursors for lignin, flavonoid pigments, protectants, and furanocoumarin phytoalexins [59]. Lignin has a positive effect on plant resistance to insects. Based on its up-regulation in the soybean defense response induced by cotton worm feeding, PAL might play a key role in cotton worm-induced resistance.

Spot 7 was identified as S-adenosylmethionine synthetase (SAMS). This protein was up-regulated at both the protein and mRNA levels (Figure 4G and 5G). Previous studies showed that increased expression of SAMS was induced by mechanical wounding, JA, insect herbivory, chilling, and salinity stress [60-63]. SAMS is a key enzyme that catalyzes the formation of S-adenosylmethionine (SAM) from methionine and ATP. SAM is a precursor of polyamines and ET. Polyamines play important roles in plant defense to a variety of environmental stresses [64,65]. ET as a plant hormone that plays an important role in induced defense. Thus SAMS might play a key role in soybean induced resistance and warrants further study.

Spot 10 was identified as ascorbate peroxidase (APX) and 20S proteasome α subunit. These proteins were up-regulated at the mRNA level (Figure 5J-1 and 5J-2). APX is an enzyme in the ascorbate-glutathione cycle. The ascorbate-glutathione cycle is an important antioxidant protection system against H_2_O_2 _generated in different cell compartments. In this study, APX may contribute to induced resistance. Overexpression of the APX gene not only enhances the plant's antioxidant capacity, but also can enhance plant tolerance to drought, heat, salt and pathogens [66,67]. In eukaryotic cells, most proteins in the cytosol and nucleus are degraded via the ubiquitin-proteasome pathway. The 26S proteasome is responsible for the degradation of damaged and misfolded proteins. A previous study showed that elicitation of defense reactions in tobacco cells by cryptogein, a proteinaceous elicitor of plant defense reactions, leads to a rapid and differential accumulation of transcripts corresponding to genes that encode defense-induced subunits of 20S proteasome [68].

In summary, all of the above proteins showed changed abundances induced by cotton worm feeding on soybean leaves. Thus the induced resistance might be a result of the combined effect of these proteins.

## Conclusions

In order to better understand the defense response induced in soybean, we investigated the changes in the soluble proteome of soybean leaves damaged by cotton worm feeding using 2-DE and MALDI-TOF-MS. A total of 10 spots changed in abundance, from which 11 proteins were identified. These proteins are indicated to be involved in multiple physiological processes, including active oxygen removal, defense signal transduction and metabolism regulation. PAL and SAMS were up-regulated at both the protein and mRNA levels. It is clear that PAL plays a key role in induced disease resistance, and SAMS can catalyzes the production of many resistance-related material. They can be strongest candidates for further functional research.

## Materials and methods

### Plant materials, growth conditions and treatments

Seeds of soybean (*Glycine max *[L].Merr.cv. Nannong 99-10) were sown in 15 cm plastic pots that contained a sterile soil mixture (top soil:sand:vermiculite, 3:2:1). The seedlings were grown in a greenhouse maintained at 30 ± 5°C, 70 ± 10% RH, and a 14 h/10 h (day/night) photoperiod with supplementary metal-halide illumination. To prevent the interference of other insects, all potted plants were covered with gauze. The potted plants were watered once daily with the same volume of water. The developmental stages of soybean plants were defined as described by Fehr et al. [69].

Treatments of cotton worm (obtained from a colony maintained on an artificial diet) feeding and untreated (control) plants were applied to soybean plants at the V4 (vegetative stage with four nodes) stage of growth. Defoliation (about 20-30%) was induced by placing three third-instar cotton worm larvae on the first trifoliate leaf for 48 h, after which the larvae were removed. The leaves of control and treated plants at four sampling times (0 h, 6 h, 12 h and 24 h) after removal of the larvae were excised for identification of induced resistance, protein extraction and RNA extraction. There were three biological replications.

### Identification of induced resistance

To evaluate soybean resistance induced by cotton worm, a dual-choice test and force-feeding experiment were conducted. In the dual-choice test, three to five leaves from each pair of experimental plants were arranged opposite each other at the margins of a porcelain dish (30 cm × 20 cm). Ten cotton worm larvae, previously starved for 24 h, were released into the middle area of the porcelain dish and allowed to feed for 12 h. Leaf area was measured by a LICOR-3000 area meter (LI-COR, Lincoln, NE). The consumed area in the control (C) and treatment (T) was used to calculate the feeding-preference index (PI), where PI = 2 T/(T + C) [70]. The PI values ranged from 0 to 2, with PI = 1 indicating no feeding preference for either control or treatment leaves, PI > 1 indicating preference for treatment leaves, and PI < 1 indicating preference for control leaves. In the force-feeding experiment, control and treatment leaves were placed in 15-cm-diameter petri dishes, then five cotton worm larvae were placed in the dish. The relative growth rate (RGR) was calculated as RGR = (W1 - W2)/W1, where W1 and W2 are the weights of five cotton worm larvae before placement in the dish and after feeding for 24 h, respectively. Statistical analysis of the data was carried out using Microsoft Excel 2000. Each experiment was repeated thrice and the mean values and SD were calculated. Single factor ANOVA was carried out using SAS.

### Total protein extraction

Total protein extraction for 2-DE was modified from the literature [39]. Frozen soybean leaves (0.2 g) were ground with liquid N_2 _in ice-cold acetone (2 ml containing 10% w/v trichloroacetic acid 20 m DTT and 100 mM PMSF) after incubation at-20°C for 1 h, the proteins were precipitated by centrifugation at 19,000 × *g *for 15 min at 4°C. The pellet was washed with 3 ml ice-cold acetone containing 20 mM DTT five times by centrifugation at 19,000 × *g *for 15 min at 4°C until no chlorophyll was present in the acetone. The protein pellet was dried *in vacuo *and resuspended in lysis buffer (7 M urea, 2 M thiourea, 4% w/v CHAPS, 65 mM DTT, 0.2% v/v carrier ampholyte [pH 3.0-10.0] and 1 mM PMSF) by sonication. The insoluble debris was removed by ultracentrifugation at 45,000 × *g *for 15 min. The protein concentration was determined by the Bradford method with BSA as the standard [71]. The protein samples were stored at -80°C until use.

### Two-dimensional electrophoresis

The 2-DE experiment was performed essentially according to the Bio-Rad instruction (USA). To proceed with isoelectric focusing (IEF), Protein (250 μg) was resuspended in lysis buffer containing 0.2% (v/v) carrier ampholyte (pH 3.0-10.0), and 0.01% bromophenol blue and rehydrated with each 17 cm, pH 3-10 nonlinear immobilized pH gradient (IPG) trip for 13 h. IEF was carried out with a Protein IEF Cell (Bio-Rad) at 20°C in a stepwise manner: 250 V for 0.5 h, 500 V for 0.5 h, 1000 V for 1 h, 2000 V for 1 h, 8000 V for 5 h and 8000 V for 55,000 V h. After IEF, the IPGs were equilibrated by shaking at room temperature in equilibration buffer (6 M urea, 2% [w/v] SDS, 30% [v/v] glycerol, 50 mM Tris-HCl [pH 8.8]) containing 1% (w/v) DTT for 20 min followed by equilibration in buffer containing 3% (w/v) iodoacetamide for another 20 min. The equilibrated IPGs were transferred to a 12% polyacrylamide gel. Electrophoresis was performed in Tris/glycine/SDS buffer on a Multiphor system (Amersham Pharmacia Biotech) according to the manufacturer's recommendations. For calibration, low-molecular weight marker proteins (Amersham Biosciences) were applied on the gel via a small piece of filter paper. After electrophoresis, the 2-DE gels were stained with silver [72]. The gels were fixed in 30% (v/v) ethanol, 10% (v/v) acetic acid for 30 min, and sensitized for 30 min in 30% (v/v) ethanol, 0.2% (w/v) sodium thiosulfate and 6.8% (w/v) sodium acetate, and then rinsed thrice in water, for 10 mimutes for each wash. Impregnated the gels with 0.25% (w/v) silver nitrate for 20 min, and then rinsed them twice for 1 min for each wash in water.

Transferred the gels in developer solution containing 2.5% (w/v) sodium carbonate and 0.02% (v/v) formaldehyde until the adequate degree of staining, then transferred them to 1.46% (w/v) ethylenediaminetetraacetic acid disodium salt stop solution for 30 minutes, last washed the gels twice in water for 10 min for each wash.

### Gel image comparison and data analysis

Spot detection, spot measurement, background subtraction and spot matching were performed specifically after silver-staining of the gels using PDQuest software. Following automatic spot detection, gel images were carefully edited. Before spot matching, one of the gel images was selected as the reference gel. The amount of a protein spot was expressed as the volume of that spot which was defined as the sum of the intensities of all pixels that made up the spot. In order to correct for variability owing to silver-staining and to reflect the quantitative variation in intensity of protein spots, the spot volumes were normalized as a percentage of the total volume of all spots present in the gel. The resulting data from image analysis were transferred to PDQuest software for querying protein spots that showed quantitative or qualitative variations. Only those with significant changes (quantitative changes more than two-fold in abundance) were used for further study. Statistical analysis of the data was carried out using Microsoft Excel 2000. The standard deviation (SD) was calculated from three spots in different gels. Gel images used for statistical analysis were obtained from three independent biological repeats.

### Protein identification and database searches

Silver-stained protein spots were excised, washed with 50% v/v acetonitrile in 0.1 M NH_4_HCO_3 _and dried in a vacuum centrifuge [73]. Gel fragments were reduced in 20 μl of 10 mM DTT with 0.1 M NH_4_HCO_3 _for 45 min at 55°C. After cooling, the DTT solution was replaced with 55 mM iodoacetamide in 0.1 M NH_4_HCO_3_. After washing, the dried gel pieces were rehydrated in 10 μl digestion buffer containing 25 mM NH_4_HCO_3 _and 12.5 ng/ml trypsin (Promega, Madison, WI, USA) and incubated at 37°C overnight. Tryptic-digested peptides were extracted. Samples were analyzed by MALDI-TOF-MS and tandem TOF/TOF MS on a time-of-flight Autolex III mass spectrometer (Bruker Daltonics, Bremen, Germany). Peptide mass maps were acquired in positive reflection mode, averaging 400 laser shots per MALDI-TOF spectrum (resolution was 15,000-20,000). The Bruker calibration mixtures were used to calibrate the spectrum to a mass tolerance within 0.1 Da.

The peak list file containing the m/z ratios of precursor ions and MS/MS fragmented ions was used in searches of the web-based search engine Mascot http://www.matrixscience.com against the most recent database in the National Center for Biotechnology Information (NCBI). The initial search used green plants as the taxon. The other parameters for the searches were: enzyme trypsin; one or two missed cleavages; variable modifications of carbamidomethyl (Cys), oxidation (Met), and pyro-Glu (Nterminal Glu); peptide tolerance of 0.1-0.5 Da; MS/MS tolerance of 0.2 Da; peptide charge of 1+ 2+; and monoisotopic. Only significant hits, as defined by the MASCOT probability analysis (*P *< 0.05), were accepted.

### Real-time quantitative RT-PCR

Total RNA was isolated from frozen soybean leaves by the TRIzol method with a total RNA Kit (Tiangen, China). Reverse transcription reactions were performed with an oligo (dT)_18 _primer and ReverTra Ace Moloney murine leukemia virus reverse transcriptase (Toyobo, Japan) according to the manufacturer's instructions. All primers were designed based on the sequences obtained using BLASTN in the NCBI database or soybean genome sequences database http://www.phytozome.net. Real-time qRT-PCR was performed on an ABI 7500 real time PCR system (Applied Biosystems, USA) using the SYBR Green Realtime Master Mix (Toyobo, Japan). Tubulin (GenBank accession no. AY907703), a constitutively expressed gene with approximately equal PCR efficiency in all samples, was used as the reference gene. Data were analyzed with SDS 2.0 software (Applied Biosystems). The primer sequences used for real-time qRT-PCR are listed in Table 2.

**Table 2:** Primer sequences used for qRT-PCR

Spot no	Protein name	Forward primer (5'→3')	Reverse primer (5'→3')
1	serine/threonine kinase related protein	GTGGCCAAGCTTCAACATAGAA	GGCTTTTGTTTGGTATGTATTCATAGA

2/9	ribulose-1,5-bisphosphate carboxylase/oxygenase large subunit	CGCGGTATTTATTTCACTCAGGAT	TCTCGGTCAGAGCAGGCATA

3	Vegetative storage Protein α	GGACAAACAGGCCGTAACAGA	CTCTCGCTGCTGTTTTGTATGAA

4	cytosolic phosphoglycerate kinase	TTGCGAAGAAATGGCGACTA	CAAAGGCACGTTCAGATCGA

5	ATP synthase beta subunit	GCGCCTGCTACGACATTTG	GGTTGGAGCATAGTTGAGGTTGA

5	malate dehydrogenase	TGCCCTTTTTGCTGATGCT	TGCCCCAAGCCCAGAAC

6	phenylalanine ammonialyase	TGCTCAAGGTTGTTGATAGGGAGTA	TCCACAAGCACTTGCCTTAGC

7	S-adenosylmethionine synthetase	GAGACATGCACCAAGACCAACA	ATTTCGCGGCATGTGTCA

8	predicted protein	AACAAGTGCAAGTTCGAATCAATG	ACGACGCCGTAGAGATCGAT

10	ascorbate peroxidase	CGCTCCTCTAATGCTCCGTTT	AATCGGCGTAGCTCAAAATAGG

10	20S proteasome alpha subunit A	TTCTTCGTTGCGCTTTTTCC	CCTCGACTCATTTTTGCCCTAA

	tubulin	GGAGTTCACAGAGGCAGAG	CACTTACGCATCACATAGCA

## Competing interests

The authors declare that they have no competing interests.

## Authors' contributions

RF designed and performed research, analyzed data and wrote the manuscript. HW participates in the design of the study. YW participate in experiment that determination of induce resistance by cotton worm feeding in soybean. DY designed research and revised the manuscript. All the authors have read and approved the final manuscript.

## References

[B1] Krishnan HB Biochemistry and molecular biology of soybean seed storage proteins New Seeds 2000 2 1 25 10.1300/J153v02n04_01

[B2] Thelen JJ Ohlrogge JB Metabolic engineering of fatty acid biosynthesis in plants Metab Eng 2002 4 12 21 10.1006/mben.2001.0204 11800570

[B3] Cui ZL Gai JY A study of leaf--feeding insect species on soybeans in Nanjing area Soybean Science 1997 16 12 20

[B4] Zhan QW Gai JY Zhang YM Sun ZD Development and expression process of inheritance of resistance to cotton worm (*prodinia litura*) in soybeans Acta Genetica Sinica 2001 28 956 963 11695268

[B5] Zhang PJ Shu JP Fu CX Zhou Y Hu Y Zalucki MP Liu SS Trade-offs between constitutive and induced resistance in wild crucifers shown by a natural, but not an artificial, elicitor Oecologia 2008 157 83 92 10.1007/s00442-008-1060-8 18491145

[B6] Agrawal AA Induced responses to herbivory and increased plant performance Science 1998 279 1201 1202 10.1126/science.279.5354.1201 9469809

[B7] Attaran E Zeier TE Griebel T Zeier J Methyl salicylate production and jasmonate signaling are not essential for systemic acquired resistance in Arabidopsis The Plant Cell 2009 21 954 971 10.1105/tpc.108.063164 19329558 PMC2671706

[B8] De Vos M Van Zaanen W Koornneef A Korzelius JP Dicke M Van Loon LC Pieterse CMJ Herbivore-induced resistance against microbial pathogens in Arabidopsis Plant Physiol 2006 142 352 363 10.1104/pp.106.083907 16829584 PMC1557608

[B9] Stotz HU Pittendrigh BR Kroymann J Weniger K Fritsche J Bauke A Mitchell-Olds T Induced plant defense responses against chewing insects. Ethylene signaling reduces resistance of Arabidopsis against Egyptian cotton worm but not diamondback moth Plant Physiol 2000 124 1007 1018 10.1104/pp.124.3.1007 11080278 PMC59200

[B10] Wu J Hettenhausen C Meldau S Baldwin IT Herbivory rapidly activates MAPK signaling in attacked and unattacked leaf regions but not between leaves of *Nicotiana attenuata* Plant Cell 2007 19 1096 1122 10.1105/tpc.106.049353 17400894 PMC1867352

[B11] Egusa M Akamatsu H Tsuge T Otani H Kodama M Induced resistance in tomato plants to the toxin-dependent necrotrophic pathogen Alternaria alternata Physiol Mol Plant Pathol 2008 73 67 77 10.1016/j.pmpp.2009.02.001

[B12] Luo Y Caldwell KS Wroblewski T Wright ME Michelmore RW Proteolysis of a negative regulator of innate immunity is dependent on resistance genes in tomato and Nicotiana benthamiana and induced by multiple bacterial effectors Plant Cell 2009 21 2458 2472 10.1105/tpc.107.056044 19671880 PMC2751963

[B13] Senthil-Nathan S Kalaivani K Choi MY Paik CH Effects of jasmonic acid-induced resistance in rice on the plant brownhopper, Nilaparvata lugens Stl (Homoptera: Delphacidae) Pestic Biochem Physiol 2009 95 77 84 10.1016/j.pestbp.2009.07.001

[B14] Bi JL Felton GW Mueller AJ Induced resistance in soybean to Helicoverpa zea: Role of plant protein quality Chem Ecol 1994 20 183 198 10.1007/BF02066000 24241708

[B15] Lin H Kogan M Influence of induced resistance in soybean on the development and nutrition of the soybean looper and the Mexican bean beetle Entomol Exp Appl 1990 55 131 138 10.1111/j.1570-7458.1990.tb01356.x

[B16] Srinivas P Danielson SD Smith CM Foster JE Cross-resistance and resistance longevity as induced by bean leaf beetle, *Cerotoma trifurcata *and soybean looper, *Pseudoplusia includens *herbivory on soybean Insect Sci 2001 1 1 5 10.1673/031.001.0501 PMC355889 15455065

[B17] Kraemer ME Rangappa M Gade W Benepal PS Induction of trypsin inhibitors in soybean leaves by Mexican bean beetle (Coleoptera: Coccinellidae) defoliation Econ Entomol 1987 80 237 241

[B18] Kessler A Baldwin IT Defensive function of herbivore-induced plant volatile emissions in nature Science 2001 291 2141 2144 10.1126/science.291.5511.2141 11251117

[B19] Reymond P Farmer EE Jasmonate and salicylate as global signals for defense gene expression Curr Opin Plant Biol 1998 1 404 411 10.1016/S1369-5266(98)80264-1 10066616

[B20] Ton J Van Pelt JA Van Loon LC Pieterse CMJ Differential effectiveness of salicylate-dependent and jasmonate/ethylene-dependent induced resistance in Arabidopsis Mol Plant Microbe Interact 2002 15 27 34 10.1094/MPMI.2002.15.1.27 11858171

[B21] Van Loon JJA de Boer JG Dicke M Parasitoid-plant mutualism: parasitoid attack of herbivore increases plant reproduction Entomol Exp Appl 2000 97 219 227 10.1046/j.1570-7458.2000.00733.x

[B22] Glazebrook J Contrasting mechanisms of defense against biotrophic and necrotrophic pathogens Annu Rev Phytopathol 2005 43 205 227 10.1146/annurev.phyto.43.040204.135923 16078883

[B23] Halitschke R Baldwin IT Antisense LOX expression increases herbivore performance by decreasing defense responses and inhibiting growth-related transcriptional reorganization in *Nicotiana attenuata* Plant J 2003 36 794 807 10.1046/j.1365-313X.2003.01921.x 14675445

[B24] Thomma BPHJ Penninckx IAMA Cammue B Broekaert WF The complexity of disease signaling in Arabidopsis Curr Opin Immunol 2001 13 63 68 10.1016/S0952-7915(00)00183-7 11154919

[B25] Howe GA Jasmonates as signals in the wound response Plant Growth Regulation 2004 23 223 237

[B26] Zavala JA Patankar AG Gase K Hui D Baldwin IT Manipulation of endogenous trypsin proteinase inhibitor production in *Nicotiana attenuata *demonstrates their function as antiherbivore defenses Plant Physiol 2004 134 1181 1190 10.1104/pp.103.035634 14976235 PMC389942

[B27] Dicke M Agrawal AA Bruin J Plants talk, but are they deaf? Trend Plant Sci 2003 8 403 405 10.1016/S1360-1385(03)00183-3 13678903

[B28] Heil M Silva Bueno JC Within-plant signaling by volatiles leads to induction and priming of an indirect plant defense in nature Proc Natl Acad Sci 2007 104 5467 5472 10.1073/pnas.0610266104 17360371 PMC1838500

[B29] Lee S Lee EJ Yang EJ Lee JE Park AR Song WH Park OK Proteomic identification of annexins, calcium-dependent membrane binding proteins that mediate osmotic stress and abscisic acid signal transduction in Arabidopsis Plant Cell 2004 16 1378 1391 10.1105/tpc.021683 15161963 PMC490033

[B30] Salekdeh GH Siopongco J Wade LJ Ghareyazie B Bennett J A proteomic approach to analyzing drought-and salt-responsiveness in rice Field Crops Res 2002 76 199 219 10.1016/S0378-4290(02)00040-0

[B31] Yao WH Biology characteristics of Prodenia litura Entomol J East China 2005 14 122 127

[B32] Ruohom ki K Hanhim ki S Haukioja E Iso-livari L Neuvonen S Niemel P Suomela J Variability in the efficacy of delayed inducible resistance in mountain birch Entomol Exp Appl 1992 62 107 115 10.1111/j.1570-7458.1992.tb00649.x

[B33] Roland J Myers JH Improved insect performance from host-plant defoliation: winter moth on oak and apple Ecol Entomol 1987 12 409 414 10.1111/j.1365-2311.1987.tb01022.x

[B34] Gibberd R Edwards PJ Wratten SD Wound-induced changes in the acceptability of tree-foliage to Lepidoptera: within-leaf effects Oikos 1988 51 43 47 10.2307/3565805

[B35] Green TR Ryan CA Wound-induced proteinase inhibitor in plant leaves: a possible defense mechanism against insects Science 1972 175 776 777 10.1126/science.175.4023.776 17836138

[B36] Karban R Carey JR Induced resistance of cotton seedlings to mites Science 1984 225 53 54 10.1126/science.225.4657.53 17775661

[B37] Underwood NC The timing of induced resistance and induced susceptibility in the soybean-Mexican bean beetle system Oecologia 1998 114 376 381 10.1007/s004420050460 28307781

[B38] Krah A Wessel R Plei ner KP Assessment of protein spot components applying correspondence analysis for peptide mass fingerprint data Proteomics 2004 4 2982 2986 10.1002/pmic.200400926 15378752

[B39] Lin YZ Chen HY Kao R Chang SP Chang SJ Lai EM Proteomic analysis of rice defense response induced by probenazole Phytochemistry 2008 69 715 728 10.1016/j.phytochem.2007.09.005 17950386

[B40] Gygi SP Rochon Y Franza BR Aebersold R Correlation between protein and mRNA abundance in yeast Mol Cell Biol 1999 19 1720 1730 10022859 10.1128/mcb.19.3.1720 PMC83965

[B41] Ideker T Thorsson V Ranish JA Christmas R Buhler J Eng JK Bumgarner R Goodlett DR Aebersold R Hood L Integrated genomic and proteomic analyses of a systematically perturbed metabolic network Science 2001 292 929 934 10.1126/science.292.5518.929 11340206

[B42] Beyer A Hollunder J Nasheuer HP Wilhelm T Post-transcriptional expression regulation in the yeast Saccharomyces cerevisiae on a genomic scale Mol Cell Proteomics 2004 3 1083 1092 10.1074/mcp.M400099-MCP200 15326222

[B43] Greenbaum D Colangelo C Williams K Gerstein M Comparing protein abundance and mRNA expression levels on a genomic scale Genome Biol 2003 4 117 124 10.1186/gb-2003-4-9-117 12952525 PMC193646

[B44] Nie L Wu G Zhang W Correlation of mRNA expression and protein abundance affected by multiple sequence features related to translational efficiency in *Desulfovibrio vulgaris*: a quantitative analysis Genetics 2006 174 2229 2243 10.1534/genetics.106.065862 17028312 PMC1698625

[B45] Canova MJ Kremer L Molle V pETPhos: a customized expression vector designed for further characterization of Ser/Thr/Tyr protein kinases and their substrates Plasmid 2008 60 149 153 10.1016/j.plasmid.2008.05.002 18597845

[B46] Wang XQ Yang PF Liu Z Liu WZ Hu Y Chen H Kuang TY Pei ZM Shen SH He YK Exploring the mechanism of Physcomitrella patens desiccation tolerance through a proteomic strategy Plant Physiol 2009 149 1739 1750 10.1104/pp.108.131714 19211702 PMC2663739

[B47] Chen LR Markhart AH Shanmugasundaram S Lin TY Early developmental and stress responsive ESTs from mungbean, Vigna radiata (L.) Wilczek, seedlings Plant Cell Reports 2008 27 535 552 10.1007/s00299-007-0488-3 18060406

[B48] Jeong RD Lim WS Kwon SW Kim KH Identification of Glycine max genes expressed in response to Soybean mosaic virus infection Plant Pathol 2005 21 47 54

[B49] Thompson GA Goggin FL Transcriptomics and functional genomics of plant defence induction by phloem-feeding insects Exp Bot 2006 57 755 766 10.1093/jxb/erj135 16495409

[B50] Sengupta S Majumder AL Insight into the salt tolerance factors of a wild halophytic rice, *Porteresia coarctata*: a physiological and proteomic approach Planta 2009 229 911 929 10.1007/s00425-008-0878-y 19130079

[B51] Wittenbach VA Purification and characterization of a soybean leaf storage glycoprotein Plant Physiol 1983 73 125 129 10.1104/pp.73.1.125 16663160 PMC1066420

[B52] Staswick PE Zhang Z Clemente TE Specht JE Efficient down-regulation of the major vegetative storage protein genes in transgenic soybean does not compromise plant productivity Plant Physiol 2001 127 1819 1826 10.1104/pp.010638 11743125 PMC133585

[B53] Berger S Mitchell-Olds T Stotz HU Local and differential control of vegetative storage protein expression in response to herbivore damage in *Arabidopsis thaliana* Physiol Plant 2002 114 85 91 10.1046/j.0031-9317.2001.1140112.x 11982938

[B54] Mason HS Mullet JE Expression of two soybean vegetative storage protein genes during development and in response to water deficit, wounding, and jasmonic acid Plant Cell 1990 2 569 579 2152178 10.1105/tpc.2.6.569 PMC159912

[B55] Xie DX Feys BF James S Nieto-Rostro M Turner JG COI1: an Arabidopsis gene required for jasmonate-regulated defense and fertility Science 1998 280 1091 1094 10.1126/science.280.5366.1091 9582125

[B56] Liu Y Ahn JE Datta S Salzman RA Moon J Huyghues-Despointes B Pittendrigh B Murdock LL Koiwa H Zhu-Salzman K Arabidopsis vegetative storage protein is an anti-insect acid phosphatase Plant Physiol 2005 139 1545 1556 10.1104/pp.105.066837 16258019 PMC1283788

[B57] Howles PA Sewalt VJH Paiva NL Elkind Y Bate NJ Lamb C Dixon RA Overexpression of L-phenylalanine ammonia-lyase in transgenic tobacco plants reveals control points for flux into phenylpropanoid biosynthesis Plant Physiol 1996 112 1617 1624 12226468 10.1104/pp.112.4.1617 PMC158095

[B58] Shadle GL Wesley SV Korth KL Chen F Lamb C Dixon RA Phenylpropanoid compounds and disease resistance in transgenic tobacco with altered expression of-phenylalanine ammonia-lyase Phytochemistry 2003 64 153 161 10.1016/S0031-9422(03)00151-1 12946414

[B59] Hahlbrock K Scheel D Physiology and molecular biology of phenylpropanoid metabolism Ann Rev Plant Biol 1989 40 347 369 10.1146/annurev.pp.40.060189.002023

[B60] Arimura G Ozawa R Nishioka T Boland W Koch T Kuhnemann F Takabayashi J Herbivore induced volatiles induce the emission of ethylene in neighboring lima bean plants Plant J 2002 29 87 98 10.1046/j.1365-313x.2002.01198.x 12060229

[B61] Cui S Huang F Wang J Ma X Cheng Y Liu J A proteomic analysis of cold stress responses in rice seedlings Proteomics 2005 5 3162 3172 10.1002/pmic.200401148 16078185

[B62] Espartero J Pintor-Toro JA Pardo JM Differential accumulation of S-adenosylmethionine synthetase transcripts in response to salt stress Plant Mol Biol 1994 25 217 227 10.1007/BF00023239 8018871

[B63] Ji W Li Y Li J Dai C Wang X Bai X Cai H Yang L Zhu Y Generation and analysis of expressed sequence tags from NaCl-treated Glycine soja BMC Plant Biol 2006 6 4 10 10.1186/1471-2229-6-4 16504061 PMC1388217

[B64] Tassoni A Franceschetti M Bagni N Polyamines and salt stress response and tolerance in *Arabidopsis thaliana *flowers Plant Physiol Biochem 2008 46 607 613 10.1016/j.plaphy.2008.02.005 18434176

[B65] Tiburcio AF Besford RT Capell T Borrell A Testillano PS Risueno MC Mechanisms of polyamine action during senescence responses induced by osmotic stress Exp Bot 1994 45 1789 1800 10.1093/jxb/45.12.1789

[B66] Sarowar S Kim EN Kim EN Kim YJ Ok SH Kim KD Hwang BK Shin JS Overexpression of a pepper ascorbate peroxidase-like 1 gene in tobacco plants enhances tolerance to oxidative stress and pathogens Plant Sci 2005 169 55 63 10.1016/j.plantsci.2005.02.025

[B67] Shi WM Muramoto Y Ueda A Takabe T Cloning of peroxisomal ascorbate peroxidase gene from barley and enhanced thermotolerance by overexpressing in *Arabidopsis thaliana* Gene 2001 273 23 27 10.1016/S0378-1119(01)00566-2 11483357

[B68] Suty L Lequeu J Lan on A Etienne P Petitot AS Blein JP Preferential induction of 20S proteasome subunits during elicitation of plant defense reactions: towards the characterization of "plant defense proteasomes" Biochem Cell Biol 2003 35 637 650 10.1016/S1357-2725(02)00386-2 12672456

[B69] Fehr WR Burmood CE Pennington DT Stage of Development Descriptions for Soybeans, Glycine Max (L.) Merrill1 Crop Sci 1971 11 929 931 10.2135/cropsci1971.0011183X001100060051x

[B70] Kogan M Feeding and nutrition of insects associated with soybeans. 2. Soybean resistance and host preferences of the Mexican bean beetle, *Epilachnavarivest* Ann Entomol Soc Am 1972 65 675 683

[B71] Bradford MM A rapid and sensitive method for the quantitation of microgram quantities of protein utilizing the principle of protein-dye binding Anal Biochem 1976 72 248 254 10.1016/0003-2697(76)90527-3 942051

[B72] Blum H Beier H Gross HJ Improved silver staining of plant proteins, RNA and DNA in polyacrylamide gels Electrophoresis 1987 8 93 99 10.1002/elps.1150080203

[B73] Hellman U Wernstedt C Gonez J Heldin CH Improvement of an "In-Gel" digestion procedure for the micropreparation of internal protein fragments for amino acid sequencing Anal Biochem 1995 224 451 455 10.1006/abio.1995.1070 7710111

